# Delites™ supplementation prevents metabolic syndrome onset and modulates gut microbiome in male Sprague Dawley rats fed on cholesterol- and fat-enriched diet: a randomized preclinical trial study

**DOI:** 10.3389/fnut.2025.1571473

**Published:** 2025-04-22

**Authors:** Juan Leonardo, Robby Hertanto, Reggie Surya, Rony Abdi Syahputra, Wardina Humayrah, Nindy Sabrina, Nurpudji Astuti Taslim, Trina Ekawati Tallei, Raymond Rubianto Tjandrawinata, Fahrul Nurkolis

**Affiliations:** ^1^Citra Deli Kreasitama Ltd., Banten, Indonesia; ^2^Increase Laboratorium Indonesia, Biomedical Campus BSD City, Tangerang, Banten, Indonesia; ^3^Department of Food Technology, Faculty of Engineering, Bina Nusantara University, Jakarta, Indonesia; ^4^Department of Pharmacology, Faculty of Pharmacy, University of North Sumatra, Medan, Indonesia; ^5^Nutrition Study Program, Faculty of Food Technology and Health, Sahid University, Jakarta, Indonesia; ^6^Division of Clinical Nutrition, Department of Nutrition, Faculty of Medicine, Hasanuddin University, Makassar, Indonesia; ^7^Department of Biology, Faculty of Mathematics and Natural Sciences, Sam Ratulangi University, Manado, Indonesia; ^8^Faculty of Biotechnology, Center for Pharmaceutical and Nutraceutical Research and Policy, Atma Jaya Catholic University of Indonesia, Jakarta, Indonesia; ^9^State Islamic University of Sunan Kalijaga (UIN Sunan Kalijaga), Yogyakarta, Indonesia; ^10^Master of Basic Medical Science, Faculty of Medicine, Universitas Airlangga, Surabaya, Indonesia; ^11^Medical Research Center of Indonesia, Surabaya, East Java, Indonesia

**Keywords:** traditional Chinese medicine, diabetes, obesity, microbiome, drugs development, *in vivo*, dyslipidemia, phytochemicals

## Abstract

**Background:**

Metabolic syndrome (MetS) is a global health concern, characterized by a combination of dyslipidemia, insulin resistance, obesity, and hypertension, significantly increasing the risk of type 2 diabetes mellitus (T2DM) and cardiovascular diseases. Gut microbiota plays a pivotal role in MetS pathophysiology, with dysbiosis exacerbating metabolic impairments. Delites™, a supplement inspired by Traditional Chinese Medicine, has shown potential in modulating gut microbiota and mitigating MetS.

**Objectives:**

This study aimed to evaluate the effects of Delites™ supplementation on metabolic health and gut microbiota composition in male Sprague Dawley rats fed a cholesterol- and fat-enriched diet (CFED).

**Methods:**

A randomized preclinical trial was conducted on 32 rats divided into four groups: control-normal, CFED, CFED+low-dose Delites™ (54 mg/kg), and CFED+high-dose Delites™ (108 mg/kg). Parameters including lipid profiles, enzymatic activity, molecular biomarkers, and gut microbiota composition were analyzed.

**Results:**

Delites™ significantly improved lipid profiles, reduced inflammation (TNF-*α*), enhanced anti-inflammatory markers (IL-10), and increased energy metabolism regulator PGC-1α. Gut microbiota modulation showed increased beneficial genera (*Bifidobacterium*, *Lactobacillus*) and reduced pathogenic *Proteus*, improving microbial diversity.

**Conclusion:**

Delites™ supplementation effectively mitigates MetS through metabolic and microbiota modulation. These findings highlight its potential for precision medicine approaches to combat metabolic disorders. Further research is needed to explore its long-term effects and translational relevance in humans.

## 1 Introduction

Metabolic syndrome (MetS) represents a global health concern, characterized by a constellation of metabolic abnormalities including dyslipidemia, insulin resistance, obesity, and hypertension (1–4). These interrelated conditions substantially increase the risk of developing type 2 diabetes mellitus (T2DM), cardiovascular diseases, and other chronic illnesses (5, 6). Recent studies have emphasized the pivotal role of gut microbiota in the pathophysiology of MetS, shedding light on how microbial dysbiosis can exacerbate metabolic impairments (7–9). Interventions targeting gut microbiota have emerged as promising strategies for mitigating the burden of metabolic disorders (9–12).

Delites™, a formulation inspired by Traditional Chinese Medicine, represents a novel approach in addressing metabolic dysfunctions. Preliminary evidence suggests that this supplement modulates gut microbiota composition, potentially restoring microbial diversity and enhancing metabolic resilience. However, the mechanisms by which Delites™ influences metabolic outcomes and gut microbiota dynamics remain underexplored, particularly in the context of high-fat and cholesterol-enriched diets—a common contributor to the global obesity epidemic. Delites™ or Chong Cao Ling Zhi Xiang Tang (13), a polyherbal formulation combining *Ganoderma lucidum* 100 mg, *Momordicae conchincinensis* Fructus 100 mg, *Paenoiae albiflora* Radix 25 mg, *Rehmania glutinosa* Radix 50 mg, *Dioscorea hypoglauca* Rhizoma 50 mg, *Corni officinalis* Fructus 50 mg, *Alismatis onentalis* Rhizoma 50 mg, *Poria cocos* 50 mg, and *Cordyceps sinensis* 25 mg, represents an innovative approach in this realm. Preliminary analyses have revealed that Delites™ contains diverse bioactive compounds (13) (Table 1), including apocynin, curcumin, and quercetin, known for their antioxidant, anti-inflammatory, and antineoplastic properties. These compounds interact with key molecular targets implicated in cervical cancer progression, such as EGFR, HSP90AA1, and MAPK1, providing a multi-modal mechanism of action (13).

**Table 1:** Metabolites profile of Delites™ via metabolomics analysis with liquid chromatography–high-resolution mass spectrometry (LC-HRMS) (13).

No. of code	Compounds name	Formula	PubChem ID
C1	Apocynin	C_9_H_10_O_3_	2214
C2	Curcumin	C_21_H_20_O_6_	969516
C3	Maltol	C_6_H_6_O_3_	8369
C4	Albiflorin	C_23_H_28_O_11_	24868421
C5	Berberine	C_20_H_17_NO_4_	2353
C6	Sachaliside 2	C_30_H_32_O_12_	44257071
C7	Nobiletin	C_21_H_22_O_8_	72344
C8	Volkenin	C_12_H_17_NO_7_	181811
C9	Cannabidivarin	C_19_H_26_O_2_	11601669
C10	Catechol	C_6_H_6_O_2_	289
C11	Daedaleanic acid A	C_31_H_46_O_4_	11179164
C12	4-Aminophenol	C_6_H_7_NO	403
C13	Farinomalein	C_10_H_13_NO_4_	44254797
C14	Piptamine	C_23_H_41_N	10664275
C15	Formononetin	C_16_H_12_O_4_	5280378
C16	Juanleoxy Fahrulanoside	C_12_H_23_NO_9_	172407454
C17	4-Prenylresveratrol	C_19_H_20_O_3_	5281725
C18	Apigenin	C_15_H_10_O_5_	5280443
C19	Quercetin	C_15_H_10_O_7_	5280343

This study investigates the efficacy of Delites™ supplementation in mitigating MetS and modulating gut microbiota in male Sprague Dawley rats subjected to a cholesterol- and fat-enriched diet. By assessing lipid profiles, enzymatic activities, biomolecular markers, and microbiota composition, this research aims to elucidate the multifaceted impact of Delites™ on metabolic and microbiome health. By addressing a critical gap in understanding the interaction between dietary supplements and gut microbiota, this study highlights the therapeutic promise of Delites™.

## 2 Materials and methods

### 2.1 Preparation and procurement of Delites™

Delites™ was obtained from Citra Deli Kreasitama Ltd. (13) and processed using previously described standardized protocols, including methods such as vacuum drying and percolation techniques, aimed at preserving the integrity of bioactive compounds. In the laboratory, the formulation was further prepared under controlled conditions, dissolved in suitable solvents such as dimethyl sulfoxide (DMSO) or buffer solutions, and stabilized for subsequent *in vitro* testing.

### 2.2 *In vivo* preclinical trial study

#### 2.2.1 Animal model and experimental setup

All experimental rats were provided unrestricted access to standard food and water *ad libitum*. A total of 32 male Sprague Dawley rats (aged 3–5 weeks) were procured from the Animal Husbandry Farm in Indonesia. The animals were housed in groups within cages under controlled laboratory conditions, maintained at a temperature of 27°C ± 2°C, and subjected to a 12-h light/dark cycle. To acclimatize to the experimental environment, the rats were kept under these conditions for 7 days prior to the intervention. All procedures adhered strictly to the Guidelines for Reporting *In Vivo* Experiments (ARRIVE) and has also been reviewed-accepted in the Preclinical Trials Study Register with number PCTE0000560. Daily observations were conducted by licensed veterinarians to monitor the animals’ welfare, assessing for signs such as reduced appetite, ruffled fur, lethargy, withdrawal behavior, hiding, or curling up. Weekly evaluations of body weight and general health markers were also performed. The sample size for the study was determined using the Federer formula (t − 1)(r − 1) ≥ 15 (14, 15), where t represents the number of treatments and r is the number of replicates or rats per treatment group. With four treatments (*t* = 4), the equation becomes (4 − 1)(*r* − 1) ≥ 15, simplifying to 3(*r* − 1) ≥ 15, and further to *r* ≥ 6. To account for potential animal losses or deaths, the sample size was increased to eight rats per treatment group, ensuring sufficient statistical power and robustness of the results.

#### 2.2.2 Experimental groups and treatments

The study employed a randomization process using a random number table for simple randomization to allocate subjects into groups (16). Blinding was implemented at multiple levels to minimize bias. Intervention providers and diet providers were not informed of the specific type of diet or feed administered to the animals. Similarly, data processors responsible for analyzing the results were blinded to the dietary or feed interventions to ensure unbiased data assessment. The rats were randomly divided into four groups, each receiving a specific dietary and treatment protocol:

- Control-Normal (CON-NORM, Group A): Standard pellet diet and water *ad libitum* (no CFED or Delites™).
- Control-Negative (CON-NEG, Group B): Cholesterol- and Fat-Enriched Diet (CFED) with water *ad libitum*.
- Low-Dose Delites™ (Delites-L, Group C): CFED supplemented with 54 mg/kg body weight of Delites™ and water *ad libitum*.
- High-Dose Delites™ (Delites-H, Group D): CFED supplemented with 108 mg/kg body weight of Delites™ and water *ad libitum*.

**Figure 1:**
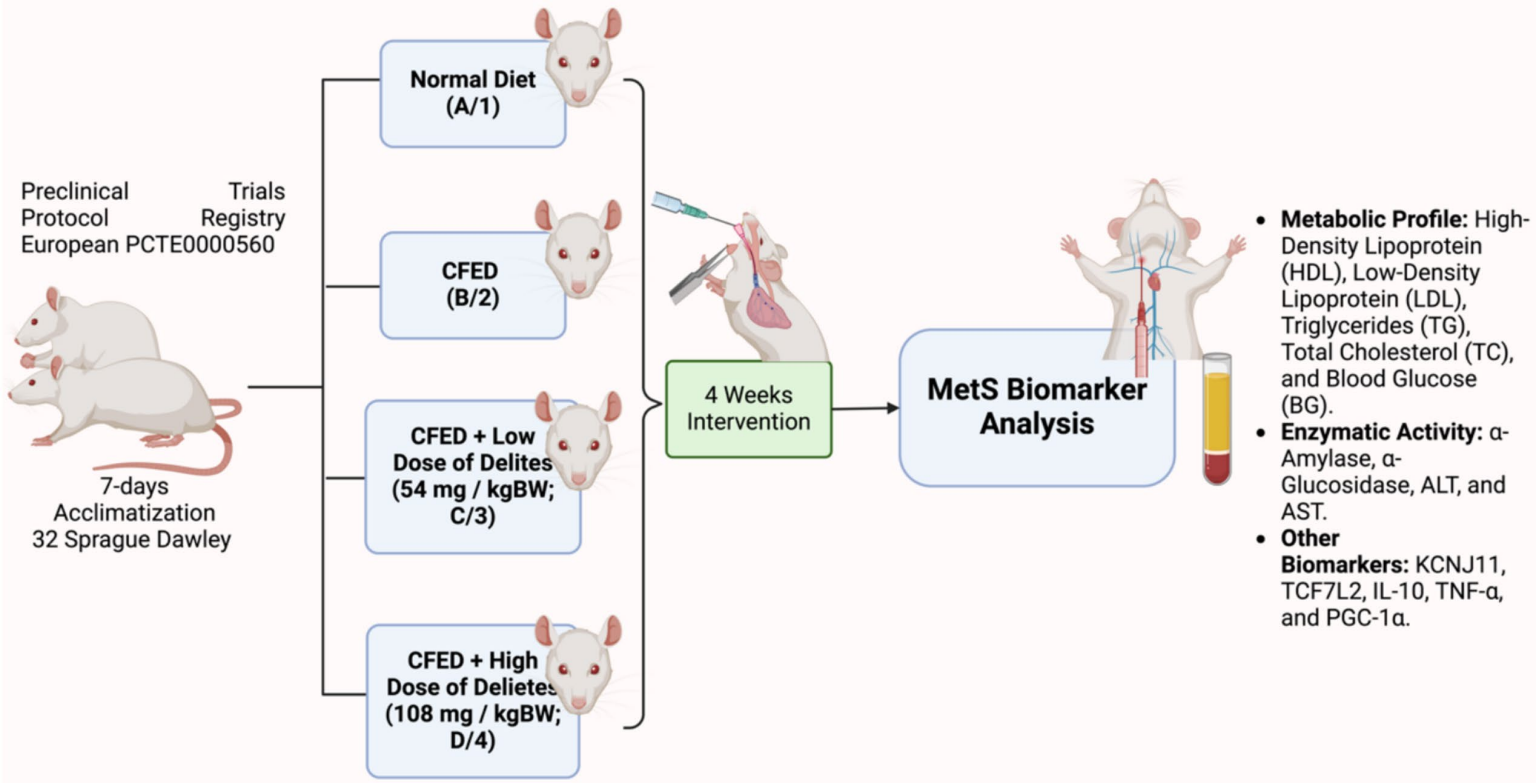
Study design of Delites™ *in vivo* preclinical trial. Created with BioRender.com.

Delites™ administration was conducted orally by certified personnel. Throughout the study, the daily intake of food and water was monitored to ensure consistency between the control and experimental groups. The dosage selection was based on the conversion factor proposed by Laurence and Bacharach (17), using a human-to-rat conversion factor of 0.018. The daily human consumption of Delites™ is 2 and 4 capsules, with each capsule containing 500 mg. This corresponds to a lower dose of 1,000 mg (2 capsules) and a higher dose of 2,000 mg (4 capsules) for humans. These values were converted using the factor, leading to the selection of the respective doses for the rat study. The detailed experimental study design can be found in Figure 1.

#### 2.2.3 Sample collection and analysis

After 4 weeks of dietary intervention, blood samples were collected following an overnight fasting period. Ketamine was administered as anesthesia, and blood was drawn from the venous sinus. Samples were transferred into sterile, anticoagulant-free tubes and allowed to coagulate at room temperature before being centrifuged at 1,000 *g* for 20 min to separate the serum.

Blood glucose and cholesterol levels were analyzed using a COBAS Integra^®^ 400 plus analyzer (Roche). Liver tissue samples were washed with 1% Phosphate Buffered Saline (PBS, pH 7.4) until clear. These samples were centrifuged (3,000 rpm, 20 min) to separate the pellet and supernatant, the latter being used for the analysis of PGC-1*α* levels. PGC-1α concentrations were quantified using the Mouse PGC-1α ELISA Kit (Sunlong Biotech Co., Ltd.).

Blood glucose, high-density lipoprotein, low-density lipoprotein, triglycerides, total cholesterol, ALT, and AST were analyzed using COBAS Integra^®^ 400 plus analyzer (Roche). Enzymatic activity assays for *α*-Amylase and α-Glucosidase were conducted using standard protocols involving spectrophotometric determination (18), where enzyme inhibition was measured based on the reduction in hydrolysis rates of specific substrates such as starch and p-nitrophenyl-*α*-D-glucopyranoside (pNPG), respectively, monitored by measuring absorbance changes at specific wavelengths (540 nm for α-Amylase and 405 nm for α-Glucosidase). Other biomarkers, such as KCNJ11 (ABIN1380468), TCF7L2 (MBS2887486), IL-10 (BMS614), TNF-α (BMS607-3), and PGC-1α (MBS776726) were quantified using ELISA kit. All procedures were carried out in accordance with the manufacturers’ instructions. Absorbance readings were obtained using microplate reader (BioTek Synergy HTX, BioTek Instruments Inc., Winooski, VT, United States). The experimental protocols for biomarker assessment followed previously established methods (14, 15).

#### 2.2.4 Dietary preparation

The Cholesterol- and Fat-Enriched Diet (CFED) consisted of 1% cholic acid, 2% pure cholesterol powder, 20% animal fat (pork oil), and 2% corn oil. These components were homogeneously mixed with standard rat chow, formed into a dough using 1,000 mL of distilled water, shaped into small pellets, and air-dried under sterile conditions. CFED batches were prepared weekly and stored at 4°C to minimize oxidation.

#### 2.2.5 Analysis of rat feces for gut microbiota

Fecal samples were stored at −80°C prior to conducting gut microbiome analysis. Intestinal bacterial genomes were extracted from these samples using the OMG Soil Extraction Kits provided by Shanghai Meiji Biopharmaceutical Technology Co., Ltd. (Shanghai, China). The V3-V4 variable regions of the 16S rRNA gene were amplified via polymerase chain reaction (PCR) using the primers 338F (5′-ACTCCTACGGGAGGCAGCAG-3′) and 806R (5′-GGACTACHVGGGTWTCTAAT-3′). Sequencing was carried out on the Illumina Miseq PE300 platform. Raw sequencing data were processed using FAST software (version 3.5.2), and sequences were trimmed with FLASH software. High-quality reads were clustered into Operational Taxonomic Units (OTUs) at 97% similarity using UPARSE software (version 7.1). Potential chimera sequences were removed through specialized search algorithms. This was achieved by comparing sequences against the Silva 16S rRNA database (Version 138, maintained by the Max Planck Institute for Marine Microbiology and Jacobs University, Bremen, Germany) with a 70% similarity threshold.

### 2.3 Data analysis and management

Multivariate ANOVA was applied to analyze several parameters, including lipid profile markers such as LDL, HDL, TG, TC, and BG; inflammatory biomarkers IL-10, TNF-*α*, and PGC-1α; enzymatic indicators α-Amylase, α-Glucosidase, ALT, AST; and molecular biomarkers KCNJ11, TCF7L2, IL-10, TNF-α, and PGC-1α in an *in vivo* experimental setting. One-way ANOVA was used to examine variations in secondary parameters, such as water intake (mL), food intake (g), food efficiency ratio (FER), and changes in body weight (initial, final, and weight gain in g) among the groups. Data were reported as mean ± standard error of the mean (SEM) with a 95% confidence interval. Statistical analyses for both *in vitro* and *in vivo* data were performed using GraphPad Prism software version 10.4.1 (Boston, MA, United States) on a MacBook/Mac Os.

## 3 Results

**Figure 2:**
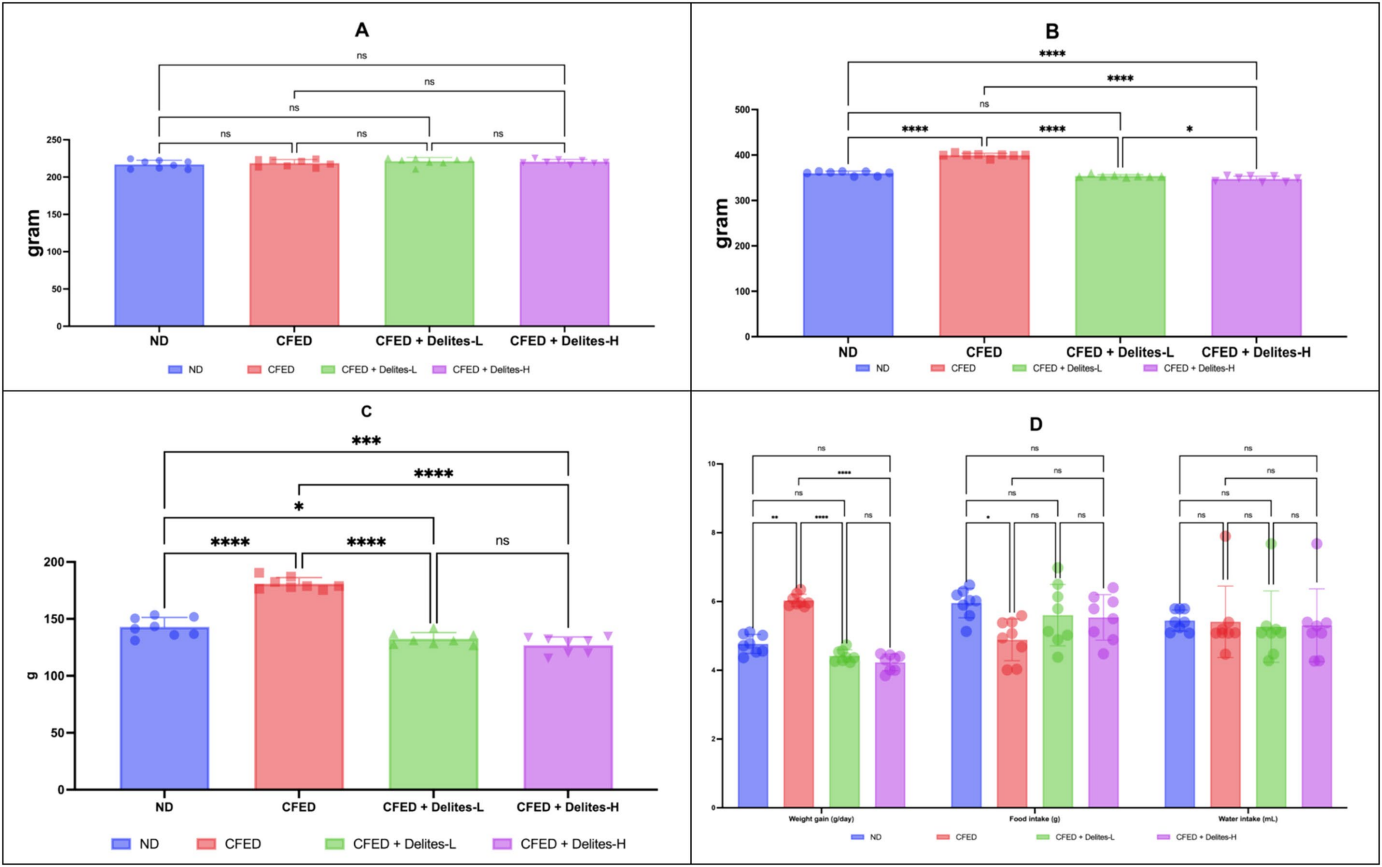
**(A)** Initial body weight in all groups. Not significant (NS) *p* > 0.05. **(B)** Final body weight in all groups. **(C)** Change in body weight (*Δ*). **(D)** Weight gain, food intake and water intake data. ****, *p < 0.05; not significant (NS) *p* > 0.05.

Initial body weights show good homogeneity as a baseline (*p* > 0.05; Figure 2A). Delites™ supplementation exhibited dose-dependent effect on weight reduction post-intervention (Figure 2B), which high-dose supplementation even shows lower weight compared to control group. The bar graph (Figure 2C) shows change in body weight (*Δ*) of rats across four experimental groups represented by blue, red, green, and purple bars. Food and water intake were similar between groups (Figure 2D).

**Figure 3:**
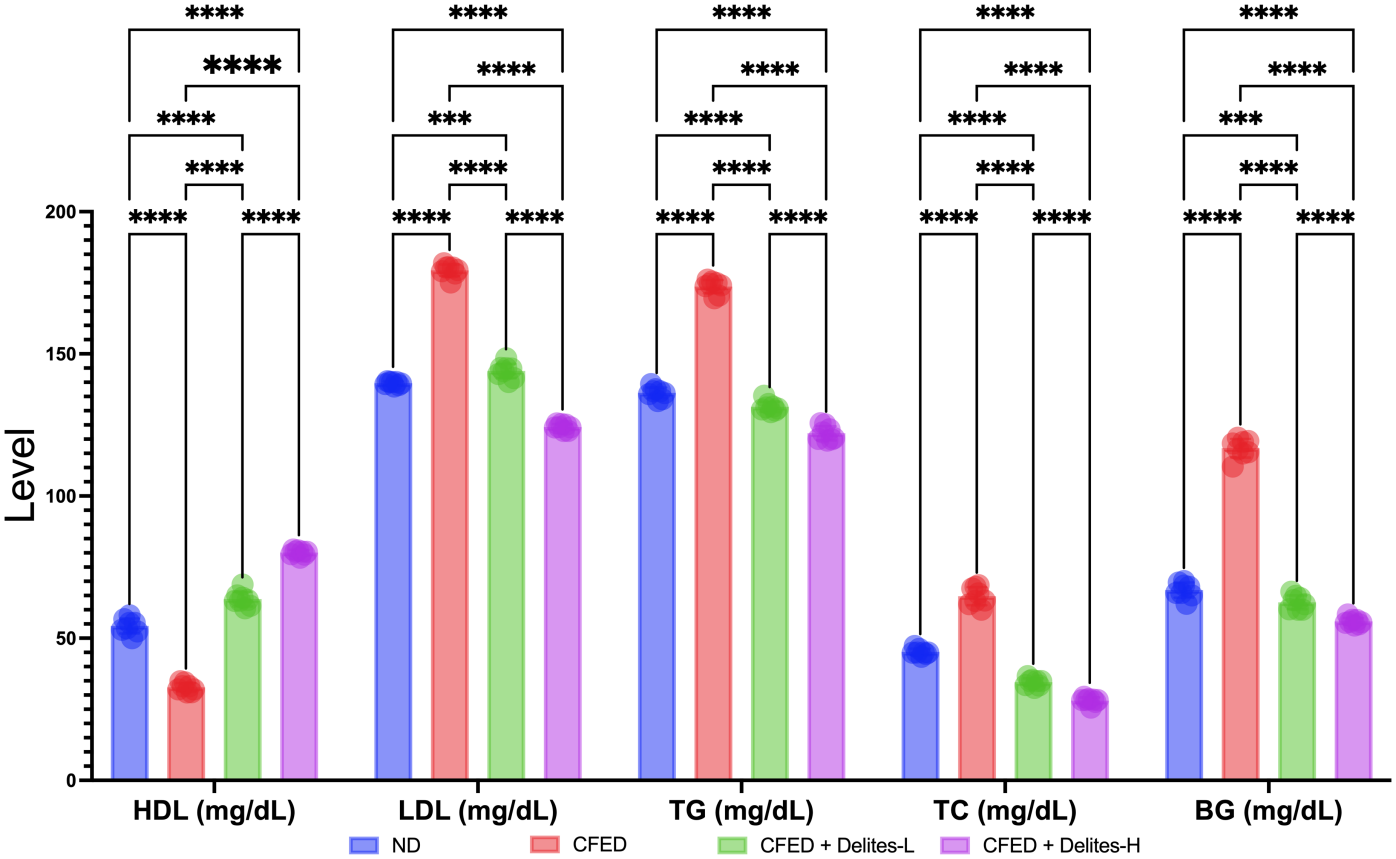
Effect of Delites™ on lipid profile and blood glucose in rats fed by CFED. HDL, High-density lipoprotein; LDL, low-density lipoprotein; TG, triglycerides; TC, total cholesterol; BG, blood glucose. ****, ***p < 0.05; not significant (NS) *p* > 0.05.

**Figure 4:**
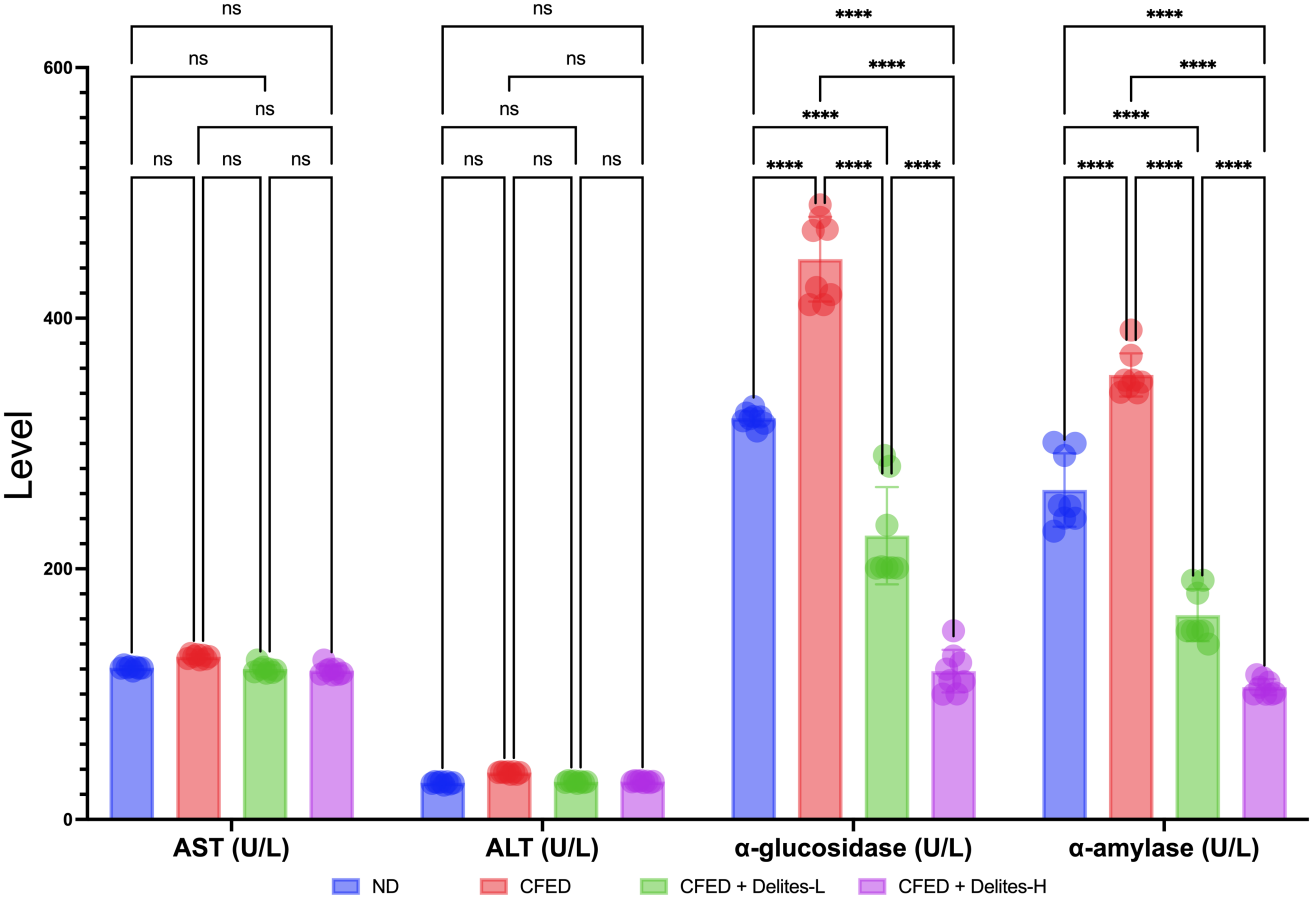
Effect of Delites™ on serum enzyme levels in rats fed by CFED. AST, aspartate transaminase; ALT, alanine transaminase; α-amylase, α-glucosidase. ****p < 0.0001; not significant (NS) *p* > 0.05.

Delites™ supplementation also shows significant dose dependent reduction of lipid and glucose profiles (Figure 3) while improving several serum enzyme profiles (Figure 4). Both Delites™ groups show significant improvement in HDL profile, while reducing the LDL, TG, TC, and blood glucose level compared to the other groups, except for the low-dose Delites™ that only exhibits protective effect against the CFED. All groups do not show any differences in AST nor any differences in ALT profile. Low and high Delites™ supplementation shows reduction on *α*-glucosidase and α-amylase levels against control and CFED groups. These results exhibited the protective effect of Delites™ in improving the regulation of the lipid and carbohydrate metabolism.

**Figure 5:**
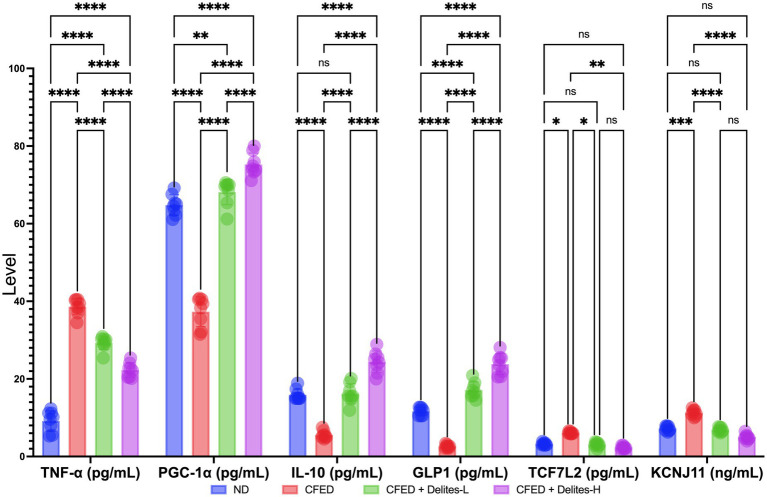
Effect of Delites™ on molecular biomarkers in rats fed by CFED. TNF-α, Tumor necrosis factor alpha; PGC-1α, Pparg coactivator-1 alpha; IL-10, interleukin-10; GLP1, glucagon-like peptide-1; TCF7L2, transcription factor 7-like 2; KCNJ11, Potassium Inwardly Rectifying Channel Subfamily J Member 11. ****, ***, **, *p < 0.05; not significant (NS) *p* > 0.05.

Molecular biomarker analysis indicates dose-dependent anti-inflammatory effect of Delites™ supplementation shown by reduction of TNF-α and increased level of IL-10; dose dependent repair on energy metabolism pathways shown by increased level of PGC-1α; dose-dependent repair on glucose metabolism shown by increased level of GLP1 and non-dose-dependent protective effect on TCF7L2 (Figure 5).

**Figure 6:**
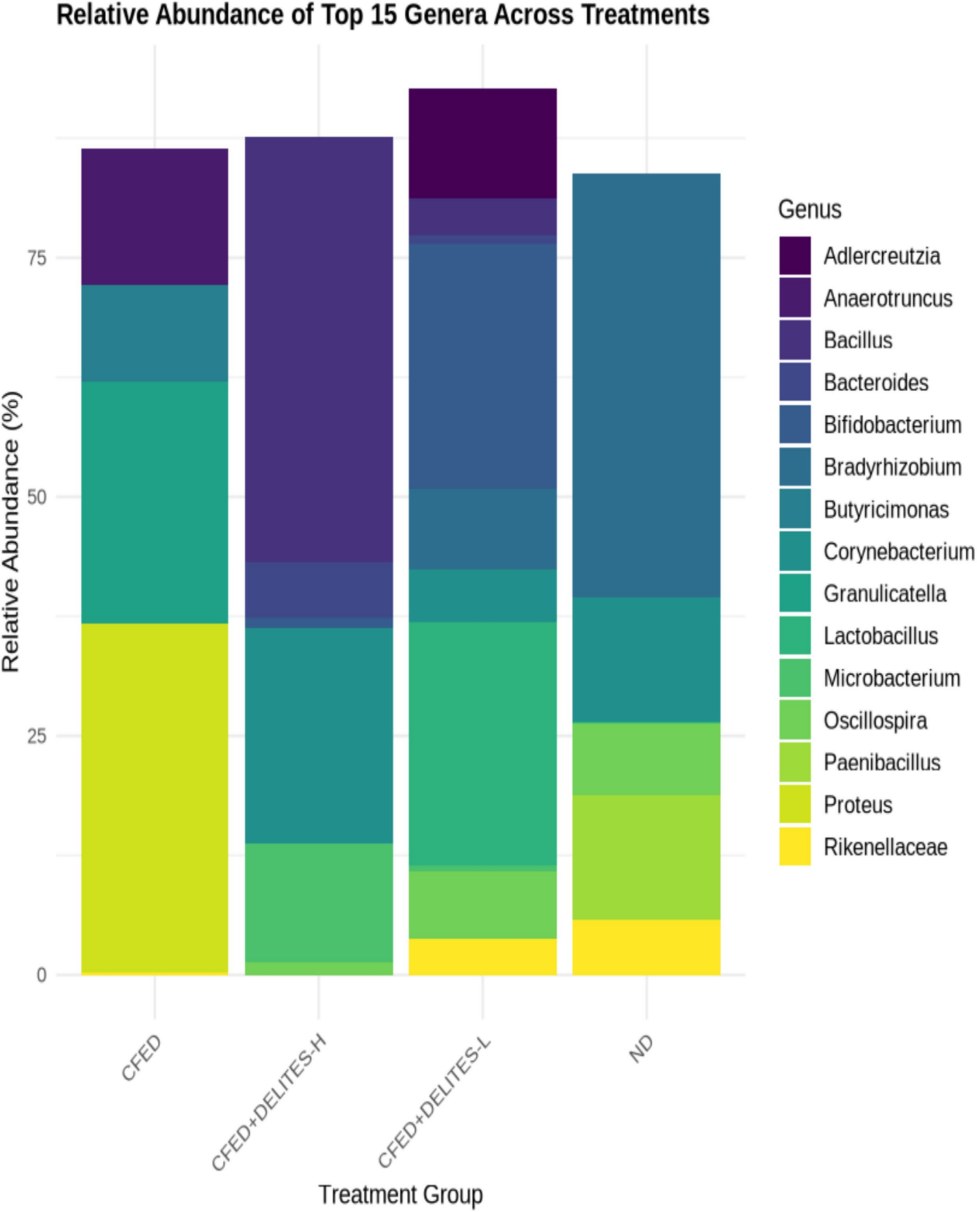
Taxonomic composition at the genus level. Each colored bar represents the percentage of each phylum or genus relative to the total microorganisms.

**Figure 7:**
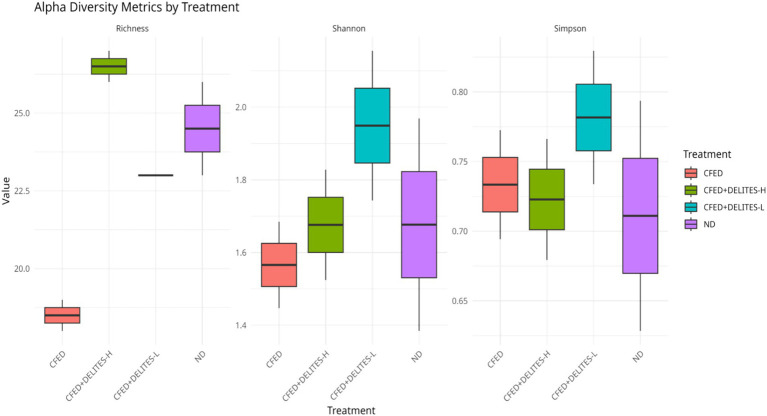
Shannon and Simpson alpha diversity index.

The gut microbiota analysis revealed that the CFED diet increased the dominance of pathogenic bacteria, such as *Proteus*, which was significantly reduced following Delites™ supplementation (Figure 6). Low-dose Delites™ supplementation enhanced the proportion of probiotic bacteria, such as *Bifidobacterium* and *Lactobacillus*, while high-dose supplementation increased *Bacillus* and *Corynebacterium*, both known for their immunomodulatory effects. Additionally, the Shannon and Simpson diversity indices indicated that the CFED+DELITES™-L group exhibited the highest microbiota diversity, suggesting a more balanced and healthier microbial profile compared to the other groups (Figure 7).

The taxonomic composition at the genus level illustrates the distribution of microorganisms within each treatment group, with each colored bar representing the relative percentage of a genus or phylum in relation to the total microbiota (Figure 6). In the group fed a high-fat and high-cholesterol diet (CFED), the genus *Proteus* dominated, accounting for 36.52%, indicating a reduction in microbial diversity. Supplementation with Delites™ led to significant changes in microbial composition. In the group receiving low-dose Delites™ (CFED+DELITES-L; Figure 6), the proportions of *Bifidobacterium* (25.60%) and *Lactobacillus* (25.43%) increased, reflecting an improved microbiota profile enriched with probiotic bacteria. In contrast, the high-dose Delites™ group (CFED+DELITES™-H) was dominated by *Bacillus* (44.46%) and *Corynebacterium* (22.63%), showing a more balanced distribution compared to the CFED group. The normal diet control group (ND) was primarily dominated by the genus *Bradyrhizobium* (44.38%), with a significant presence of *Corynebacterium* (13.03%). These findings highlight that Delites™ supplementation, both at low and high doses, effectively reduced the dominance of pathogens like *Proteus* and promoted the presence of beneficial bacteria, resulting in a healthier and more diverse microbiota profile.

**Figure 8:**
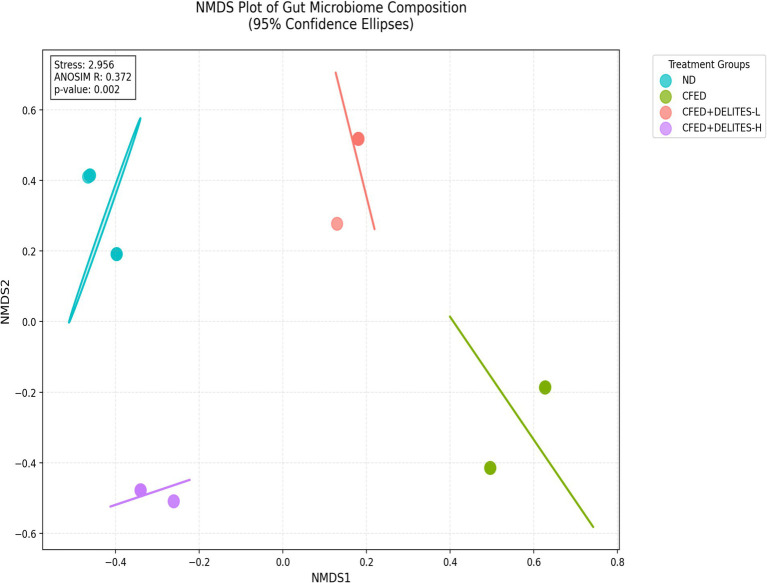
Non-metric multidimensional scaling (NMDS) plot of all samples using the Bray-Curtis resemblance matrix by ANOSIM analysis.

**Figure 9:**
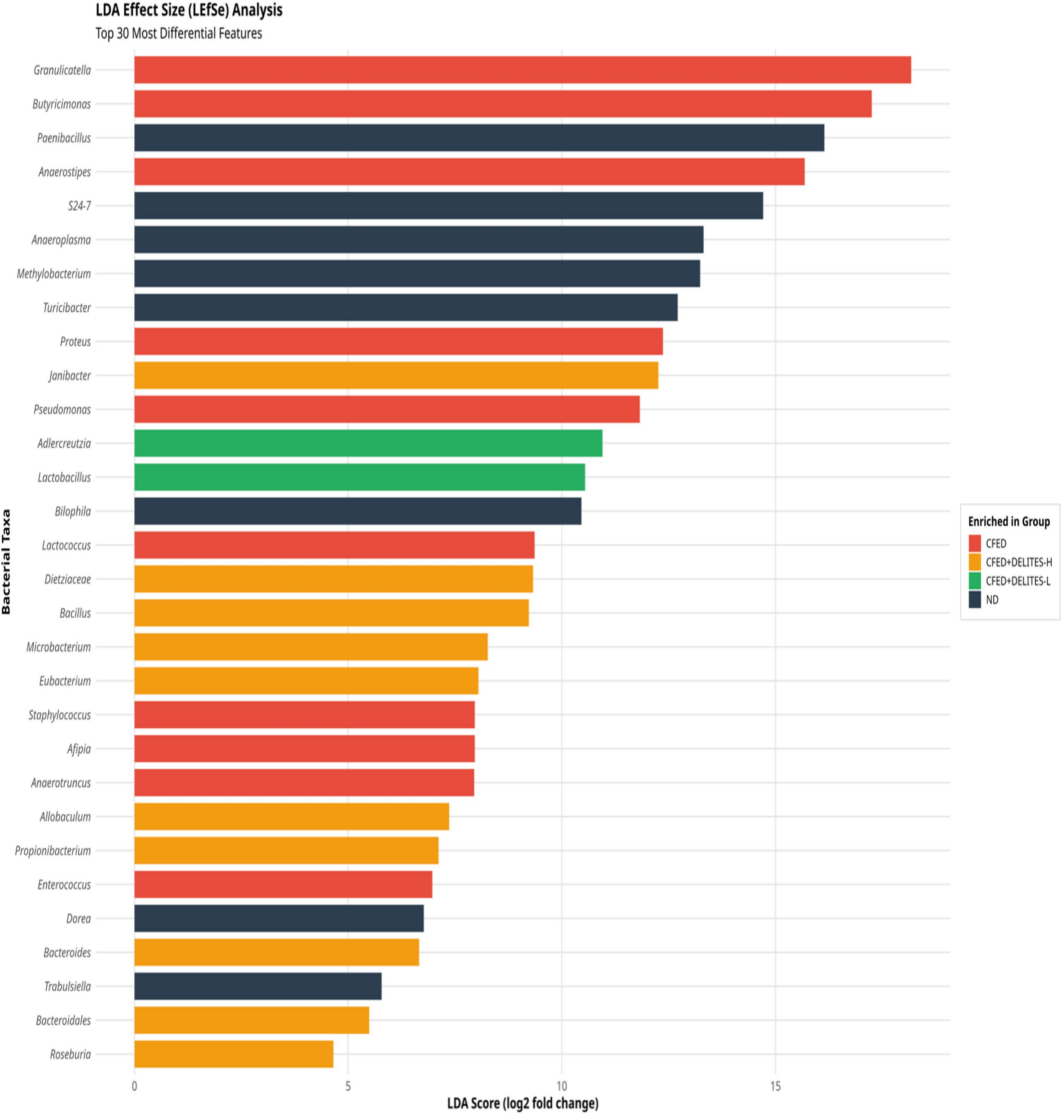
Linear discriminant analysis (LDA) effect size (LEfSe) analyses of gut microbiota according to diet at the genus level.

Statistical analysis using ANOSIM revealed significant differences in microbiota composition between the groups (R = 0.372, *p* = 0.002), further supporting the observation that Delites™ can significantly modulate gut microbiota (Figure 8). Additionally, LEfSe analysis identified bacteria such as *Granulicatella* and *Anaerostipes*, which were dominant in the CFED group and associated with carbohydrate metabolism (Figure 9). In contrast, Delites™ supplementation induced a shift in the microbiota composition toward a healthier and more balanced profile.

**Figure 10:**
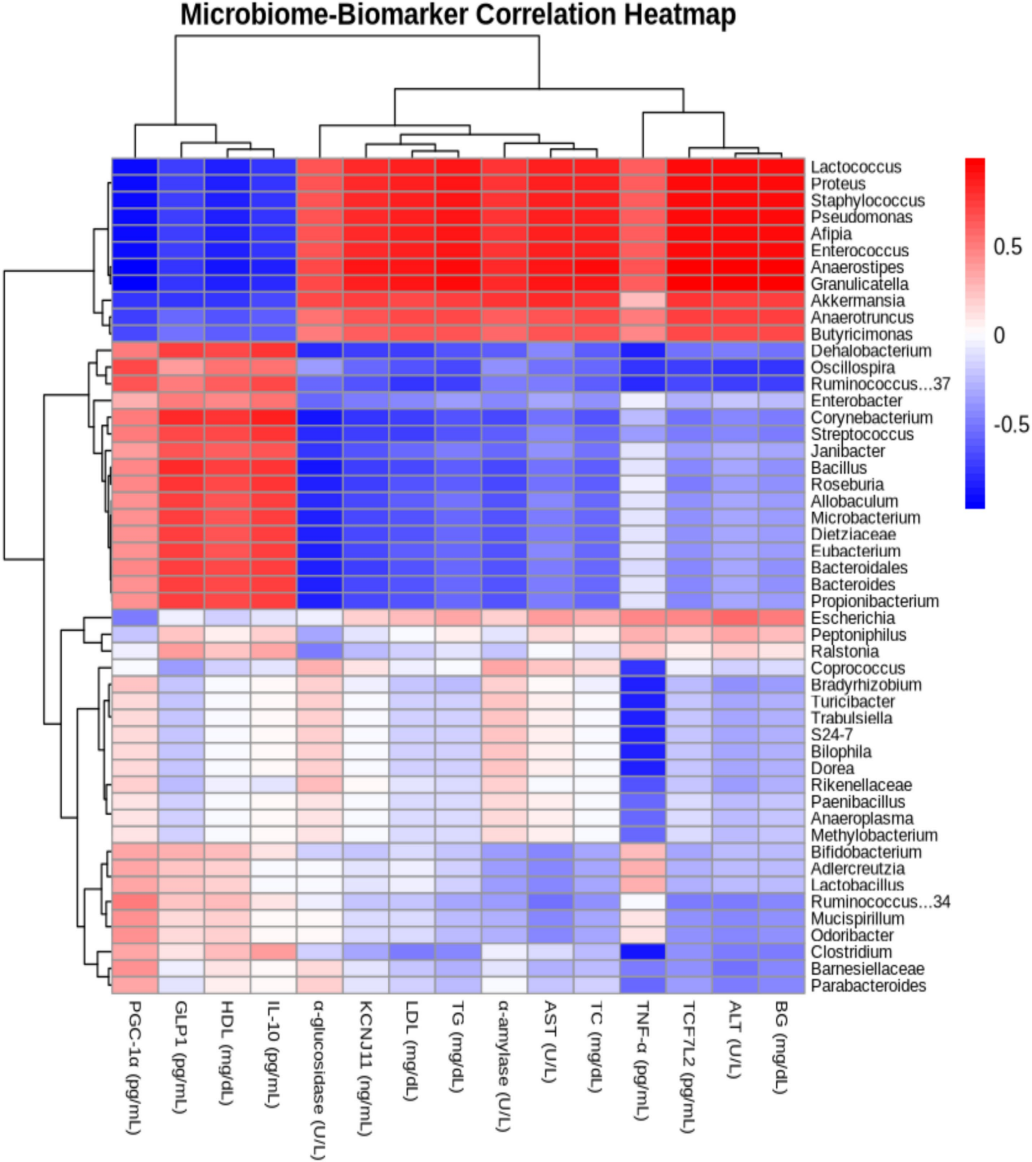
Heatmap illustrating the Pearson correlation coefficients between gut microbiome composition and blood metabolic profiles in rats, providing insights into the relationships between specific microbial taxa and key metabolic biomarkers.

The correlation analysis between gut microbiota and biomarkers revealed that Anaerostipes showed a positive correlation with ALT, blood glucose, and TCF7L2, but a negative correlation with PGC-1α, a key regulator of energy metabolism (Figure 10). Similarly, Granulicatella demonstrated a positive correlation with ALT and blood glucose. These findings highlight a strong relationship between gut microbiota composition and metabolic regulation, underscoring the potential of Delites™ as an effective intervention for metabolic disorders. Overall, this study emphasizes the protective effects of Delites™ against metabolic syndrome, achieved through gut microbiota modulation and improvement of metabolic biomarkers.

## 4 Discussion

**Figure 11:**
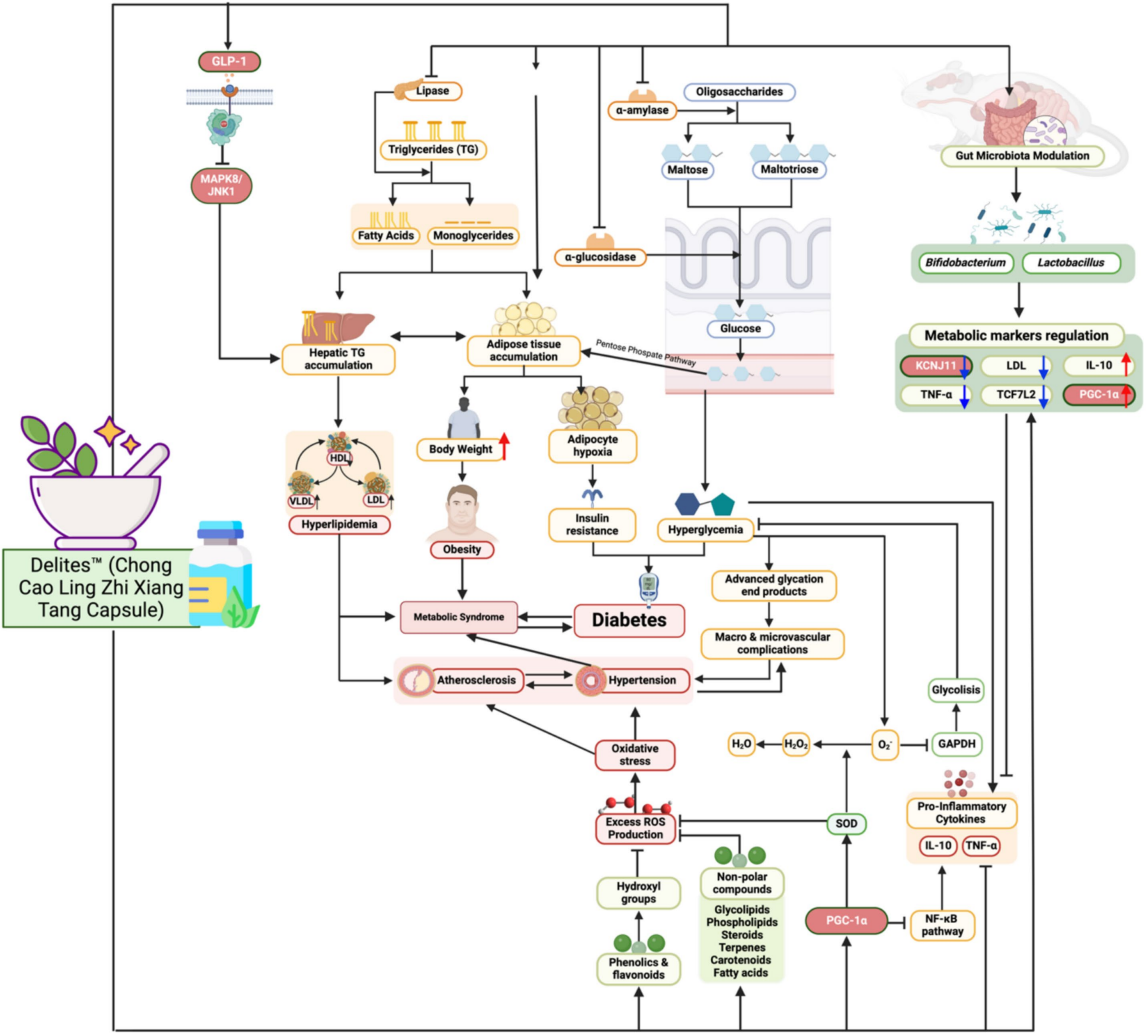
Biomechanism of Delites™ in Mitigates Metabolic Syndrome and Modulation Gut Microbiome. Created with BioRender.com.

This study demonstrates that Delites™ supplementation attenuated the metabolic disruptions induced by a cholesterol- and fat-enriched diet, as evidenced by improvements in lipid profiles, blood glucose levels, and inflammatory markers. Specifically, supplementation led to a dose-dependent reduction in LDL, TG, TC, and TNF-*α*, alongside increased HDL, IL-10, and PGC-1α levels. These findings suggest that Delites™ exerts both anti-inflammatory and metabolic regulatory effects, consistent with modulation of the gut microbiota (Figure 11). These results support the hypothesis that Delites™ has potential as a multifaceted intervention for addressing metabolic syndrome (MetS) through integrated metabolic and microbiological mechanisms (13). Figure 11 highlights the multifaceted mechanisms through which Delites™ (Chong Cao Ling Zhi Xiang Tang Capsule) exerts its beneficial effects on metabolic health. This traditional Chinese medicinal formulation appears to target several critical nodes in the regulation of lipid and glucose metabolism, inflammation, and mitochondrial function.

One of the central pathways modulated by Delites™ is the inhibition of lipogenesis and triglyceride synthesis, as evidenced by downregulation of protein and enzyme such as SREBP-1c and ACC1 (19, 20) (Figure 11). By suppressing lipogenic gene expression, Delites™ reduces hepatic triglyceride accumulation, thereby attenuating hepatic steatosis, which is a major contributor to insulin resistance. Moreover, Delites™ promotes lipolysis and fatty acid *β*-oxidation, shifting the metabolic balance toward energy expenditure rather than storage.

Importantly, Delites™ also enhances insulin sensitivity through multiple routes (Figure 11). It reduces systemic inflammation by downregulating key pro-inflammatory cytokines such as TNF-*α*, IL-6, and IL-1β (21). This anti-inflammatory effect is supported by activation of PGC-1α, a master regulator of mitochondrial biogenesis and antioxidant response. Enhanced mitochondrial function further contributes to improved glucose utilization and reduced reactive oxygen species (ROS) generation.

Furthermore, Delites™ appears to positively influence the gut microbiota composition, increasing the abundance of beneficial commensals while suppressing pro-inflammatory microbial species (Figure 11). This microbial modulation likely leads to increased production of short-chain fatty acids (SCFAs) such as butyrate and acetate, which have been associated with improved intestinal barrier integrity, reduced endotoxemia, and enhanced GLP-1 secretion. Elevated GLP-1, in turn, supports glycemic control by promoting insulin secretion and inhibiting glucagon release (22).

Specifically, Delites™ supplementation positively impacted lipid profiles, including reductions in LDL, triglycerides, total cholesterol, and blood glucose levels, along with an increase in HDL levels. These effects suggest protective mechanisms against cardiovascular disease risks commonly associated with MetS (23–25). These benefits are likely linked to elevated levels of PGC-1α, a critical regulator of energy metabolism that plays a pivotal role in lipid and glucose homeostasis (26–29).

In addition, Delites™ supplementation improved liver enzyme parameters (ALT and AST), indicating protection against hepatotoxicity induced by an unhealthy diet (30, 31). This effect was reinforced by gut microbiota modulation, where Delites™, at both low and high doses, reduced the dominance of pathogenic species such as *Proteus* while increasing the proportion of probiotic bacteria like *Bifidobacterium* and *Lactobacillus*. These findings suggest that Delites™ not only restores microbial diversity but also shifts the microbiota composition toward a healthier profile (32–34).

Microbial diversity indices (alpha-diversity), such as Shannon and Simpson indices (35–37), revealed higher diversity in the CFED+Delites™ groups compared to CFED-only groups, indicating a restorative effect of Delites™ on gut microbiota balance. Further NMDS and LEfSe analyses highlighted significant shifts in microbiota patterns, with probiotic bacteria associated with healthy metabolism becoming more dominant (38).

Moreover, correlations between microbiota and metabolic biomarkers provided additional mechanistic insights. For instance, *Anaerostipes*, which was negatively correlated with PGC-1α and positively with ALT, underscores the connection between microbiota and energy metabolism as well as liver health (39–41). Delites™ intervention successfully reduced the prevalence of *Anaerostipes*, which may contribute to enhanced energy metabolism and reduced inflammation.

These findings confirm the potential of Delites™ as a therapeutic agent leveraging the gut microbiome to mitigate MetS. By simultaneously improving metabolic parameters and modulating gut microbiota, Delites™ offers a holistic approach to managing MetS.

These findings, while preclinical, suggest a plausible mechanism in which Delites™ restores microbiota balance and metabolic homeostasis, which should be further validated in clinical settings.

### 4.1 Strengths of the study

This study possesses several significant strengths. First, its multidimensional approach, encompassing the evaluation of metabolic biomarkers, gut microbiota profiles, and physiological parameters, provides a comprehensive understanding of Delites™’ effects on metabolic syndrome. Second, rigorous methodology involving randomization and blinding ensures the validity of the results, while robust statistical analyses enhance the reliability of data interpretation. Furthermore, the translational relevance of this study is a crucial aspect, as its findings lay the groundwork for the development of microbiota-based interventions for humans, particularly for more effective management of metabolic syndrome.

### 4.2 Limitations of the study

Nonetheless, this study has some limitations that warrant consideration. As a preclinical study, the use of Sprague Dawley rats as a model may not fully represent human responses to Delites™ supplementation. The relatively short duration of the study, only 4 weeks, limits the understanding of the long-term effects of this intervention. Additionally, the study focused exclusively on the effects of Delites™ on a high-fat and high-cholesterol diet, leaving the potential impacts on other dietary patterns unexplored. Finally, although biomarker and microbiota analyses were conducted, deeper investigations into the molecular mechanisms underlying Delites™’ effects on metabolic syndrome are necessary to strengthen the existing evidence.

## 5 Conclusion

This study comprehensively demonstrates that Delites™ supplementation holds significant potential for mitigating metabolic syndrome through a multidimensional approach encompassing improvements in metabolic parameters, reduction of inflammation, and modulation of gut microbiota. Delites™ not only lowers LDL, triglycerides, total cholesterol, and blood glucose levels but also enhances HDL levels, highlighting its protective role against cardiovascular risks associated with high-fat and high-cholesterol diets. Additionally, the modulation of gut microbiota by Delites™, including increased probiotic bacteria such as *Bifidobacterium* and *Lactobacillus* and reduced dominance of pathogens like *Proteus*, underscores its potential to restore healthy microbiota balance.

With its gut microbiota-based approach, Delites™ offers a novel paradigm for holistic and root-cause-focused management of metabolic syndrome. These findings not only pave the way for the development of nutrition-based interventions for humans but also strengthen the relevance of Delites™ as a potential therapeutic agent in precision medicine. However, to maximize the translation of these findings to human populations, further studies with longer durations and diverse dietary contexts are needed. This research thus provides a crucial foundation for future exploration of Delites™ as an innovative solution to the challenges of metabolic syndrome in the modern era.

## Data Availability

The original contributions presented in the study are included in the article/supplementary material, further inquiries can be directed to the corresponding author.
